# Effects of the application of a prolonged combined intervention on body composition in adolescents with obesity

**DOI:** 10.1186/s12937-020-00570-8

**Published:** 2020-05-27

**Authors:** Teodoro Durá-Travé, Fidel Gallinas-Victoriano, María Urretavizcaya-Martinez, Lotfi Ahmed-Mohamed, María Jesús Chueca-Guindulain, Sara Berrade-Zubiri

**Affiliations:** 1 Department of Pediatrics, School of Medicine, University of Navarra, Pamplona, Spain; 2 Department of Pediatrics, Navarra Hospital Complex, Avenue Irunlarrea, 431008 Pamplona, Spain; 3 Navarra Institute for Health Research (IdisNA), Pamplona, Spain

**Keywords:** Fat free mass, Fat mass index, Multidisciplinary obesity treatment, Body composition, Waist circumference

## Abstract

**Background:**

The aim of this study is to describe the effects of a prolonged dietary-behavioral-physical activity intervention (24 months) on body composition in a group of adolescents with obesity.

**Methods:**

Longitudinal study in 196 individuals with obesity (86 boys and 110 girls) aged 10.1–14.9 years that completed a prolonged combined intervention (24 months). Values for weight, height, skinfold thickness, waist circumference, BMI, body fat, fat mass index (FMI) and fat-free mass index (FFMI) were registered or calculated. A good response to treatment was reported when a BMI z-score reduction of greater than or equal to 0.5 units of the initial value occurred after 24 month of follow up.

**Results:**

A good response after 24 months of follow-up reached 58.2% (*n* = 114). In boys with obesity and BMI status improvement, weight z-score, BMI z-score, body fat, and FMI significantly decreased (*p* < 0.05). In girls with obesity and BMI status improvement, weight z-score, BMI z-score, waist circumference, waist z-score, body fat and FMI significantly decreased (*p* < 0.05). In both sexes the height and FFMI increased significantly (*p* < 0.05). The multiple logistic regression analysis showed that girls and younger age were associated with BMI status improvement; concurrently, the place of residence (urban or rural) and degree of obesity were not associated with BMI status improvement.

**Conclusion:**

The application of long-term combined strategies in the treatment of childhood obesity seems to be effective. As BMI decreases, a reduction in fat mass is also detected, with evident sexual dimorphism, in the absence of changes in fat-free mass and, consequently, in longitudinal growing.

## Introduction

The prevalence of excess body weight (overweight and obesity) in children has progressively increased in recent decades, reaching 22.5% of the adolescent population in our region (Navarra, Spain) [1]. Its treatment is complex and to date has disappointing results; however, interventions related to child obesity that combine steps on feeding, physical activity, sedentary lifestyle and changes in standards of conduct, as well as an active family implication, are more effective in the reduction of body mass index (BMI) than isolated interventions [2]. On the other hand, even when intensive interventions show better results on a short-term basis, the duration of its positive results is rather limited [3–5].

The effectiveness of the interventions on child obesity does not lie in the achievement of a hasty loss weight in the shortest time possible, but rather in the acquisition of healthy habits by the patients and their family environment [6]. Therefore, the long-term combined interventions would turn to be the most adequate in order to consolidate healthy dietary habits and lifestyle, and, consequently, to reduce the excess body fat; however, there are few studies on the effectiveness of long-term interventions in the treatment of child obesity [7, 8].

Even though body mass index is the most frequently used anthropometric measurement in the diagnosis and follow up of child obesity [8–10], it provides limited information, since it does not allow the discrimination of changes in the composition of the different body compartments: fat mass and fat-free mass [11]. Since the main objective of the treatment of child obesity consists in the reduction of the percentage of body fat mass, several authors advocate the use of the fat mass index (FMI) due to its higher sensibility to detect changes in body fat [12–14]. In other way, waist-height ratio (WtHR) is a marker of visceral fat and its reduction could have some clinical interest since it is a modifiable cardiovascular disease risk factor [15, 16].

Dual-energy X-ray absorptiometry (DEXA) is considered a reference technique for the assessment of body composition; however, the complexity of installation and management and high cost makes it unfeasible for daily clinical practice. On the other hand, the fat mass index calculated using skin fold measurement shows a good correlation with total body fat evaluated by DEXA [14, 17–19].

The objective of this study is to describe the changes in several nutritional indices: BMI, WtHR, FMI and fat-free mass index (FFMI) using anthropometric techniques in a group of adolescents with obesity enrolled in an extensive combined intervention (24 months) to reduce excess body fat.

## Methods

### Participants

This was a longitudinal study (convenience sample) conducted in 196 obese individuals (86 boys and 110 girls) aged 10.1 to 14.9 years that fulfilled a combined dietary-behavioral-physical activity intervention program during a period of 24 months (clinical evaluation was performed every 3 months). All patients involved in the study were Caucasian and showed pubertal changes (Tanner stages: II-V). All the participants and their parents or legal guardians were offered adequate information, fulfilled informed consent, and were subsequently included in the combined intervention program that was carried out from January, 2015 to December, 2018. This study was approved by the Ethics Committee for Human Investigation at our institution (in accordance with the ethical standards described in the 1964 Declaration of Helsinki and later amendments). The exclusion criteria were: obesity secondary to genetic, metabolic or endocrine disease. Residence was categorized as urban or rural (more or less than 10.000 inhabitants, respectively).

### Anthropometric measurements

The standardised protocol that was used for the anthropometric measurements has been previously published [6]. The following anthropometric measurements were registered in the first consultation and every 3 months: weight, height, body mass index (BMI), skinfold thickness (biceps, triceps, subscapular and suprailiac) and waist circumference.

Weight and height measurements were taken with participants wearing only undergarments and barefoot. Weight was measured using an Año-Sayol scale (reading interval 0 to 120 kg and a precision of 100 g), and height was measured using a Holtain wall stadiometer (reading interval 60 to 210 cm, precision 0.1 cm). BMI was calculated according to the following formula: weight (kg)/height ^2^ (m).

Skinfold thickness values were measured with an accuracy of 0.1 mm on the left side of the body with Holtain skinfold calipers (CMS Weighing Equipment, Crymych, United Kingdom). The percentage of total body fat, fat mass (kg) and fat-free mass (kg) was calculated using the equations reported by Slaughter et al., adjusted for sex and age [20]. In the same way, the FMI and the fat free mass index (FFMI) were calculated using the following formulas: fat mass (kg) / height^2^ (m)., and free fat mass (kg) / height^2^ (m), respectively.

Waist circumference (WC) was registered using a tape measure placed on a horizontal line equidistant from the last rib and the iliac crest, and the WtHR was calculated according to the formula: waist (m)/ height^2^ (m). Measurements were performed by the same trained individual.

The z-score values for the weight, height, BMI, skinfold thickness and WC were calculated using the program *Aplicación Nutricional*, from the Spanish Society of Pediatric Gastroenterology, Hepatology and Nutrition (*Sociedad Española de Gastroenterología, Hepatología y Nutrición Pediátrica*, available at http://www.gastroinf.es/nutritional/). The graphics from Ferrández et al. (Centro Andrea Prader, Zaragoza 2002) were used as reference charts [21].

The z-score value for BMI allowed establishing the following BMI status:

- Normal: z-score between − 1.0 (15th percentile) and + 1.0 (85th percentile).
- Overweight: z-score > 1.0 (85th percentile).
- Obesity: z-score > 2.0 (97th percentile).
- Severe obesity: z-score > 3.0 (99th percentile).

### Combined dietary-behavioral-physical activity intervention

The combined intervention has been previously explained [6]. The central idea of the program corresponds to the following maxim: “the child becomes skinny keeping a stable weight because he/she is growing” and it includes nutritional education, a nutritional intervention, the promotion of physical activity and healthy lifestyles and self-monitoring of body weight (weekly registration of weight).

The acquisition of basic practical and theoretical skills that enables self-monitoring was mandatory in order to be included in this study. A multidisciplinary team (pediatrician, nurse and dietitian) educated the patients and their families on nutrition, synchronizing the education and the first visit. The contents of these structured sessions (nutritional value of the different food groups, food pyramid, physical activity, etc.) were personalized according to the characteristics of each patient and family and continuous guidance was provided for all of them. The program was developed or extended depending on the needs of the patient in subsequent visits.

The approach to weight maintenance is accomplished by means of a diversified and well balanced diet for the whole family with no strict restrictions or immediate or exaggerated weigh loss. The Mediterranean diet, adapted to family customs or the preferences of the patients, was our dietary model. It was mandatory to ensure five daily meals, with the requirement that meal schedules were respected. The participants were instructed to avoid eating in between meals and to increase the time of intake (eating slowly and adequately chewing the food).

In addition, an individualized scheme to increase physical activity was proposed to every participant and consisted of a daily, regulated (60 min) free-choice activity (swimming, walking, cycling, martial arts, etc.) and an increase in daily activity (such as walking up the stairs rather than using the elevator, walking, helping in house tasks, etc.).

Every family was given a leaflet with general recommendations on usual diet, physical activity (sports and home activity) and a healthy lifestyle.

A good response to treatment (BMI status improvement) was reported when a BMI z-score reduction of greater than or equal to 0.5 units of the initial value occurred after 24 months of follow up; otherwise, it was considered a failure to treatment [7, 22].

### Statistical analysis

Results are displayed as percentages (%) and means (M) with corresponding standard deviations (SD). The statistical analysis (descriptive statistics, Student’s t test, analysis of variance, Chi-square test, and multiple logistic regression analysis) was performed using the program Statistical Packages for the Social Sciences version 20.0 (SPSS, Chicago, IL, USA). Statistical significance was assumed when *p*-value was < 0.05.

## Results

Table 1 shows and compares mean values of anthropometric characteristics registered in individuals of both sexes before the combined intervention. Mean values of weight, BMI, BMI z-score, waist, waist z-score, WtHR, triceps z-score and subscapular z-score were significantly higher in boys (*p* < 0.05). Mean values of suprailiac z-score and body fat were significantly higher in girls (*p* < 0.05). There were no significant differences in mean values of age, weigh z-score, height z-score, biceps z-score, FMI and FFMI between individuals of both sexes.

**Table 1:** Anthropometric characteristics before combined intervention in both sexes (M ± SD)

Item	Boys (*n* = 86)	Girls (*n* = 110)	*P*-value*
Age (y)	11.9 ± 1.4	11.6 ± 1.6	0.331
Weight (kg)	73.1 ± 15.4	66.6 ± 16.8	0.003
Weight z-score	3.3 ± 1.1	3.1 ± 1.1	0.134
Height (cm)	156.2 ± 12.8	152.8 ± 10.8	0.026
Height z-score	0.9 ± 1.0	1.05 ± 0.9	0.234
BMI (Kg/m^2^)	29.4 ± 3.7	27.8 ± 4.0	0.004
BMI z-score	3.4 ± 1.0	2.9 ± 1.0	0.010
Waist (cm)	96.3 ± 9.1	88.3 ± 11.1	0.001
Waist z-score	2.7 ± 0.98	2.1 ± 0.8	0.001
WtHR	0.6 ± 0.1	0.5 ± 0.1	0.001
Skinfold thickness			
Biceps z-score	2.8 ± 1.2	2.8 ± 1.3	0.857
Triceps z-score	3.1 ± 0.8	2.4 ± 0.8	0.001
Subscapular z-score	3.4 ± 1.0	2.5 ± 0.9	0.001
Suprailiac z-score	4.3 ± 1.1	5.1 ± 1.2	0.001
Body fat (%)	37.2 ± 3.7	39.6 ± 3.4	0,001
FMI (kg/m^2^)	10.6 ± 1.5	10.9 ± 1.4	0.076
FFMI (kg/m^2^)	18.4 ± 2.6	16.9 ± 2.7	0.023

(*) Student’s t-test

*BMI* Body mass index, *WC* Waist circumference, *WHR* Waist to height ratio, *FMI* Fat mass index, *FFMI* Fat-free mass index

The percentage of participants who showed an improvement in BMI status after 12 months of follow-up was 53.1% (*n* = 104), and there were statistically significant differences (*p* < 0.001) between girls (63.6%, *n* = 70) and boys (39.5%, *n* = 34). The percentage of participants who improved BMI status after 24 months of follow-up was 58.2% (*n* = 114), and revealed statistically significant differences (*p* < 0.019) between girls (*n* = 72, 65.5%) and boys (*n* = 42, 48.8%). In contrast, 29.6% (*n* = 58) of the participants did not improve BMI status after 12 and 24 months. Within the period comprised between 12 and 24 months of follow up, 17.3% (*n* = 34) of participants improved BMI status, whereas 12.2% (*n* = 24) showed a deterioration. This means, 70.2% (*n* = 80) of participants who improved BMI status after 24 months had already showed an improvement after 12 months.

Table 2 shows and compares mean values of anthropometric characteristics registered at the beginning of the study and after 12 and 24 months of follow-up in boys with obesity and BMI status improvement and with no BMI status improvement. In patients with BMI status improvement, mean values of weight z-score, BMI z-score, skinfold thickness, body fat, and FMI significantly decreased (*p* < 0.05) during the follow-up period. There were no significant differences in mean values of weight, height z-score, BMI, waist, WC z-score and WtHR throughout the combined intervention. In contrast, in patients with no BMI status improvement, mean values of weight, BMI, BMI z-score, waist, waist z-score, body fat, and FMI significantly increased (*p* < 0.05) during the follow-up period. There were no significant differences in mean values of WtHR and skinfold thickness throughout the combined intervention. The mean values of height and FFMI significantly increased (*p* < 0.05) whether they had improved their BMI status or not.

**Table 2:** Anthropometric characteristics throughout combined intervention in boys with obesity (M ± SD)

	With BMI status improvement (*n* = 42)				With no BMI status improvement (*n* = 44)			
	Baseline	12 mo	24 mo	*P*-value*	Baseline	12 mo	24 mo	*P*-value*
Age (y)	12.0 ± 1.8	12.9 ± 1.9	14.0 ± 1.9	0.001	11.8 ± 1.3	12.8 ± 1.3	13.6 ± 1.5	0.001
Weight (kg)	72.3 ± 18.3	73.4 ± 18.7	72.7 ± 18.5	0.393	73.9 ± 15.6	82.7 ± 17.9	89.8 ± 16.9	0.001
Weight z-score	3.1 ± 1.5	2.9 ± 1.4	2.1 ± 1.3	0.007	3.6 ± 1.1	3.7 ± 1.3	3.8 ± 1.3	0.730
Height (cm)	158 ± 14.5	163.1 ± 13.7	167.3 ± 12.4	0.037	156.5 ± 11.1	162.6 ± 10.8	168.3 ± 11.3	0.001
Height z-score	0.7 ± 1.4	0.6 ± 1.1	0.6 ± 0.9	0.746	0.7 ± 1.1	0.8 ± 1.2	0.8 ± 1.1	0.228
BMI (Kg/m2)	29.1 ± 4.1	29.1 ± 5.2	27.5 ± 5.1	0.513	29.8 ± 3.3	30.9 ± 3.5	32.5 ± 3.7	0.002
BMI z-score	3.3 ± 1.1	3.0 ± 1.4	2.4 ± 1.2	0.006	3.4 ± 0.91	3.8 ± 1.1	3.9 ± 1.1	0.037
Waist (cm)	96.1 ± 13.3	96.9 ± 13.1	94.5 ± 12.1	0.944	96.5 ± 8.4	100 ± .8.8	103.6 ± .9.2	0.011
Waist z-score	2.8 ± 1.2	2.6 ± 1.1	2.4 ± 1.4	0.332	2.7 ± 0.7	3.2 ± 0.9	3.2 ± 0.9	0.016
WtHR	0.6 ± 0.1	0.6 ± 0.1	0.6 ± 0.1	0.082	0.6 ± 0.1	0.6 ± 0.1	0.6 ± 0.1	0.653
Skinfold thickness								
Biceps z-score	2.8 ± 1.2	1.5 ± 0.8	1.1 ± 1.3	0.001	2.8 ± 1.3	2.8 ± 0,9	2.9 ± 1.1	0.418
Triceps z.score	3.1 ± 0.7	2.5 ± 0.9	2.1 ± 1.1	0.001	2.9 ± 0.9	2.9 ± 0.8	3.1 ± 0.9	0.438
Subescapular z-score	3.3 ± 1.1	2.6 ± 0.9	2.2 ± 1.2	0.001	3.4 ± 0.9	3.4 ± 1.1	3.5 ± 1.0	0.526
Suprailiac z-score	3.9 ± 1.1	3.6 ± 0.9	2.9 ± 1.3	0.002	4.5 ± 1.1	4.7 ± 0.8	4.7 ± 0.6	0.612
Body fat (%)	36.7 ± 3.7	35.3 ± 3.2	31.8 ± 5.1	0.001	37.7 ± 3.8	38.3 ± 2.5	39.7 ± 2.4	0.003
FMI (kg/m2)	10.8 ± 1.6	10.3 ± 1.7	9.1 ± 1.8	0.006	10.6 ± 1.3	11.4 ± 1.6	12.6 ± 1.4	0.009
FFMI (kg/m2)	18.2 ± 2.9	18.7 ± 2.9	19.2 ± 2.4	0.012	18.4 ± 2.3	19.7 ± 2.5	19.5 ± 2.6	0.043

(*) ANOVA

*BMI* Body mass index, *WHR* Waist-to-height ratio, *FMI* Fat mass index, *FFMI* Fat-free mass index

Table 3 shows and compares mean values of anthropometric characteristics registered at the beginning of the study and after 12 and 24 months of follow-up in girls with obesity and BMI status improvement and with no BMI status improvement. In patients with BMI status improvement, mean values of weight z-score, BMI z-score, waist, waist z-score, WtHR, skinfold thickness, body fat, and FMI significantly decreased (*p* < 0.05) during the follow-up period. There were no significant differences in mean values of weight, height z-score and BMI throughout the combined intervention. In contrast, in patients with no BMI status improvement, mean values of weight, body fat and FMI significantly increased (*p* < 0.05) during the follow-up period. There were no significant differences in mean values of weight z-score, height z-score, BMI, BMI z-score, waist, waist z-score, WtHR and skinfold thickness throughout the combined intervention. The mean values of height and FFMI significantly increased (*p* < 0.05) whether they had improved their BMI status or not.

**Table 3:** Anthropometric characteristics throughout combined intervention in girls with obesity (M ± SD)

	With BMI status improvement (*n* = 72)				With no BMI status improvement (*n* = 38)			
	Baseline	12 mo	24 mo	*P*-value*	Baseline	12 mo	24 mo	*P*-value*
Age (y)	10.8 ± 1.5	12.1 ± 1.5	13.2 ± 1,6	0.001	12.4 ± 1.4	13.5 ± 1.3	14.3 ± 1.4	0.001
Weight (kg)	59.1 ± 9.9	61.9 ± 8.3	61.4 ± 7.3	0.339	75.6 ± 16.9	81.6 ± 16.8	87.5 ± 16.5	0.037
Weight z-score	2.9 ± 0.9	2.4 ± 0.9	1.5 ± 0.7	0.001	3.3 ± 1.3	3.3 ± 1.5	3.5 ± 1.4	0.963
Height (cm)	149.4 ± 6.3	154 ± 4.8	158.3 ± 4.8	0.001	158 ± 8.5	162 ± 8.4	163.6 ± 8.7	0.029
Height z-score	0.8 ± 0.7	0.8 ± 0,9	0.6 ± 0,9	0.165	0.8 ± 1.1	0.8 ± 1.2	0.9 ± 1.4	0.885
BMI (Kg/m2)	26.4 ± 3.5	26.9 ± 3.3	26.2 ± 3.7	0.837	29.9 ± 4.3	30.8 ± 4.7	32.4 ± 4.4	0.157
BMI z-score	2.9 ± 1.0	2.3 ± 0.8	1.4 ± 0.7	0.001	3.0 ± 0.9	3.1 ± 1.4	3.2 ± 1.1	0.911
Waist (cm)	85.1 ± 9.1	86.5 ± 7.7	81.1 ± 7.1	0.018	95.5 ± 11.8	98.7 ± 11.6	99.2 ± 10.6	0.552
Waist z-score	1.8 ± 0.7	1.6 ± 0.7	1.2 ± 0.7	0.001	2.5 ± 1.1	2.7 ± 1.3	2.8 ± 1.3	0.524
WtHR	0.6 ± 0.1	0.6 ± 0.1	0.5 ± 0.1	0.001	0.6 ± 0.1	0.6 ± 0.1	0.6 ± 0.1	0.542
Skinfold thickness								
Biceps z-score	2.6 ± 1.3	1.5 ± 1.2	0.7 ± 0.6	0.001	3.3 ± 1.1	3.2 ± 1.1	3.3 ± 1.1	0.813
Triceps z-score	2.2 ± 0.7	1.6 ± 0.7	1.3 ± 0.6	0.001	2.7 ± 0.9	2.7 ± 0.8	2.8 ± 0.9	0.284
Subscapular z-score	2.5 ± 0.9	1.9 ± 0.8	1.4 ± 0.7	0.001	2.5 ± 0.7	2.8 ± 0.9	2.9 ± 0.8	0.141
Suprailiac z-score	4.9 ± 1.3	4.4 ± 1.3	3.2 ± 1.3	0.001	5.2 ± 0.8	5.2 ± 0.9	5.2 ± 0.9	0.334
Body fat (%)	39.9 ± 3.9	37.1 ± 4.8	34.1 ± 3.2	0.001	39.1 ± 2.1	39.3 ± 2.4	40.6 ± 2.5	0.014
FMI (kg/m2)	10.9 ± 1.3	10.5 ± 1.4	10.0 ± 1.2	0.039	11.1 ± 1.6	11.6 ± 1.5	12.7 ± 1.6	0.037
FFMI (kg/m2)	16.1 ± 2.6	16.7 ± 1.9	16.9 ± 2.1	0.030	18.1 ± 2.4	19.1 ± 2.8	19.6 ± 2.1	0.055

(*) ANOVA

*BMI* Body mass index, *WtHR* Waist-to-height ratio, *FMI* Fat mass index, *FFMI* Fat-free mass index

**Fig. 1:**
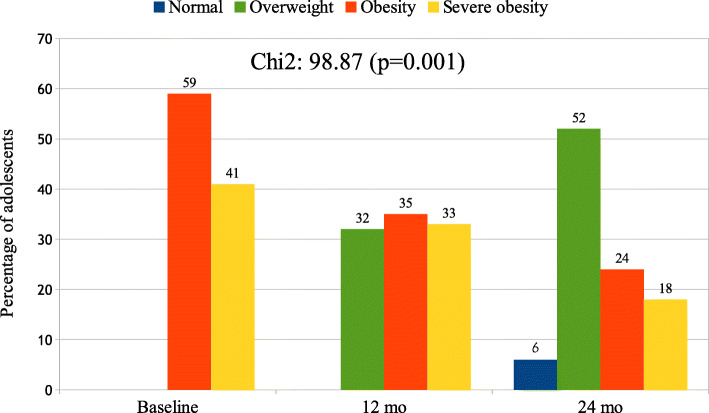
Changes in BMI status (shown as percentage) throughout combined intervention of all participants (boys and girls) with BMI status improvement

Figure 1 depicts and compares the BMI status (shown as percentage) between the beginning of the study and after 12 and 24 months of follow-up of all participants (boys and girls) in this study with BMI status improvement. Obesity and severe obesity BMI status significantly decreased and overweight BMI status significantly increased (*p* < 0.001) during the combined intervention. In boys, at the beginning of the study, severe obesity BMI status was 58%, and it was 27% after 24 month follow-up; in girls, at the beginning of the study, severe obesity BMI status was 31%, and was 12% at the end of the study (no significant differences between sexes).

**Fig. 2:**
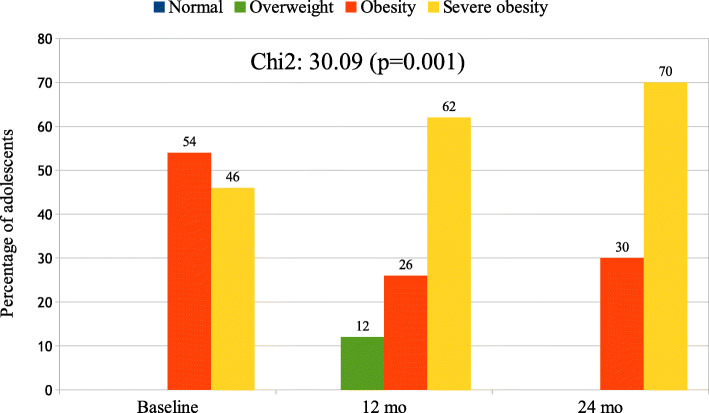
Changes in BMI status (shown as percentage) throughout combined intervention of all participants (boys and girls) with no BMI status improvement

Figure 2 depicts and compares the BMI status (shown as percentage) between the beginning of the study and after 12 and 24 months of follow-up of all participants (boys and girls) in this study with no BMI status improvement. Obesity BMI status significantly decreased and severe obesity BMI status significantly increased (*p* < 0.001) during the combined intervention. In boys, at the beginning of the study, severe obesity BMI status was 58% and increased to 90% after 24 month follow-up; in girls, at the beginning of the study, severe obesity BMI status was 31%, and it was 39% at the end of the study (*p* < 0.05, between sexes).

The multiple logistic regression analysis for the presumed factors related to BMI status improvement is represented in Table 4. Girls were associated with BMI status improvement at 12 and 24 months of follow-up, and the younger age (age groups of 10–11 and 11–12 years) were exclusively associated with BMI status improvement at 24 months of follow-up. Residence (urban or rural) and degree of obesity were not associated with BMI status improvement.

**Table 4:** Multiple logistic regression analysis for presumed factors related to BMI status improvement

Characteristics	BMI improvement at 12 mo OR (95% CI) P-value	BMI improvement at 24 mo OR (95% CI) P-value
Sex		
Boys	Referent	Referent
Girls	2.8 (1.47–5.19) 0.02	1.9 (1.1–3.47) 0.03
Age group		
14–15 years	Referent	Referent
12–13 years	0.8 (0.54–2.62) 0.767	2.3 (1.14–4.61) 0.019
10–11 years	1.3 (0.64–2.42) 0.503	3.7 (1.73–8.04) 0.001
Residence		
Urban	Referent	Referent
Rural	1.6 (0.89–2.82) 0.111	1.6 (0.91–2.91) 0.096
BMI		
Severe Obesity	Referent	Referent
Obesity	1.7 (0.98–3.07) 0.058	1.3 (0.72–2.26) 0.403

## Discussion

The main outcome of this descriptive study was that application of combined and long-term strategies in adolescents with obesity by multidisciplinary teams that, in addition, include parental active implication, seems to be satisfactory. In fact, it has been observed that prolonged combined intervention (24 months) allows, in a large proportion, the maintenance and/or improvement of previously acquired changes in body compartments (at 12 months). Despite this, it is important to emphasize why these type of strategies should be started as soon as possible and, of course, the importance of intensifying periodic controls in males owing to their more frequent tendency to be refractory to treatment.

The design of the combined intervention in which these adolescents were included took into account that the effectiveness in child and adolescent obesity largely depends on the provisioning of nutritional education to the patient and the family environment. This education should include dietary changes and restrictions, as well as an increase in daily physical and quotidian activity; self-control should not be forgotten, since motivating the patient is critical so as to get good results. In other words, it is not just a matter of getting a rushed weight loss in a short period of time, since the aphorism “the child gets skinny by keeping weight since he/she is growing …” is still valid [6, 23], but rather the development of psycho-affective adherence to dietary habits and healthy lifestyles and, as a consequence, the inclusion of these changes as habits by the patient and the family.

The analysis of the follow up period revealed that 58.2% of the participants had an improvement in BMI status (with respect to the beginning of the intervention) after 24 months, whereas only 29.6% of them did not show any improvement in BMI status after 12 and 24 months. In between 12 and 24 months, 70.2% of participants maintained this improvement in BMI status (reached at 12 months) and 17.3% of participants even upgraded this BMI status, although 12.2% of them deteriorated it. In fact, even though the totality of participants at the beginning of the intervention were classified, according to BMI, as obese and severe obese, and 24% of participants had a nutritional classification of overweight after 12 months, this percentage raised to 32% by the end of the intervention and even 4% were considered in normal status. This means, the extension of the intervention up to 24 months could be considered as satisfactory, since it seems to reinforce the acquisition of healthy habits in a majority of participants who had previously upgraded BMI after 12 months of follow up. Therefore, we could consider, with cautious optimism, that the lengthening of this kind of programs might lead to even better results.

Updated systematic reviews on the effectiveness of combined long-term interventions (24 months) in the treatment of adolescents with obesity are scarce, and show wide methodological and outcome heterogeneity and, therefore, do not facilitate drawing of clear-cut conclusions [4, 8, 24–26]. However, all the studies analyzed show that a decrease in BMI was achieved with improvement in body composition [7, 8, 27–30] and cardiovascular risk factors [27, 28, 30, 31], but without having previously determined what would be considered a good response to treatment, as established in this study.

In this study, BMI was applied for the classification and monitoring of the nutritional status of the adolescents who were included. However, although it may be useful to define obesity [7, 9, 14, 32], it grants limited information since it does not allow to discern the proportional constitution of the different body compartments: fat mass and fat-free mass [11, 12, 33–35]. In addition, this limitation becomes increasingly evident in adolescence when a series of physiological changes in body composition take place [36, 37], and they might lead to a wrong estimation of weight increase as excess body fat [38, 39] if only BMI is used. Hence, since interventions on childhood obesity intend to reduce the excess of body fat without negatively affect fat-free mass and, consequently, longitudinal growth, it would be more accurate to track the changes in the fat mass and fat-free mass indices. In fact, several authors advocate the use of the fat mass index (FMI) in contrast to the BMI in order to diagnose and monitor childhood obesity, owing to the higher sensibility to detect changes in body fat [14, 19, 36]. The assessment of subcutaneous fat by skin fold measurements allows monitoring the changes that will potentially take place in fat tissue in patients after interventions designed to reduce excess body fat. The fat mass index calculated using skin fold measurement shows a good correlation with total body fat evaluated by DEXA [14, 17–19]. This is one of the main arguments for using these indicators in this study in order to assess the evolutionary changes in body compartments.

The analysis of participants who upgraded BMI status along the 24 months of follow up confirmed that the aphorism used as reference in this study guaranteed a progressive improvement in BMI status. In fact, as a direct consequence of the lack of changes in mean values of weight and, simultaneously, the increase of mean values of height along the follow up, the BMI z-score progressively decreased in comparison to the initial values. We should warn that, at the same time BMI z-score decreases, a series of changes in body compartments occur in both sexes. On one hand, a progressive decrease in the percentage of fat mass, and, consequently, a significant decrease in FMI is detected; on the other hand, the FFMI progressively increases also in both sexes, what could explain, to a great extent, that growing up in this patients was not affected during the period of the combined intervention. Obviously, in patients with no BMI status improvement, mean values of weight, BMI z-score, WtHR, percentage of body fat and FMI significantly increased during the follow-up period.

Waist circumference, and specially WtHR, identified central obesity in children and adolescents [15, 40], and is strongly associated with cardiovascular disease risk factors [16, 41, 42]. The analysis of the evolution of waist circumference mean values -as a sign of visceral fat-, from the beginning to the end of the follow up period, showed an evident sexual dimorphism. While a significant decrease in waist circumference and WtHR is detected during follow up in girls, there were no significant changes in boys. This may indicate that BMI status improvement is associated with a parallel decrease in visceral fat more easily in obese girls adolescents; this means, it seems like sexual dimorphism in fat distribution, characteristic of obesity in adult age, initiates in this period of life. This eventuality could have some clinical interest since it is a modifiable cardiovascular disease risk factor.

The analysis of factors presumably associated to BMI status improvement (sex, age group, place of residence and degree of obesity) confirms that female gender and a younger age showed a higher tendency to experience BMI status improvement throughout the combined intervention. Nevertheless, the location of the family residence (urban or rural areas) and the level of obesity were not associated with BMI status improvement. This finding would support the hypothesis that the negative socio cultural connotations related to girl obesity in our environment encourages girls individuals to fulfill the requirements of the program and, in this way, it would be determining in the better therapeutic response compared to boys. Furthermore, the lower age we intervene, the greater is the chance to implement and consolidate healthy habits in adolescents with obesity.

An important limitation of our study is that although every 3 months the basic theoretical and practical skill were reviewed, and the practical difficulties in the application of the combined dietary-behavioral-physical activity intervention were analyzed and intended to correct, there was no strict monitoring of caloric intake and / or daily physical activity. Nor have other variables been recorded that could, to some extent, condition the results, such as the parents’ BMI, parental education, socioeconomic status, etc. Consequently, it has not been possible to perform a regression analysis with these variables. Another limitation is that the reasons of failure to treatment have not been analyzed. It is possible to think that participants with a good response to treatment might have a special degree of motivation for dietary and lifestyle changes, especially in girls, which would determine the good response to this combined intervention. Nevertheless, we may have neither sufficiently motivated all participants nor involved their families in the study.

## Conclusions

The application of an extensive combined dietary-behavioral-physical activity intervention has a positive effect in obese adolescents. As BMI decreases, a reduction in fat mass is also detected, with evident sexual dimorphism, in the absence of changes in fat-free mass and, consequently, in longitudinal growing. Finally, we should emphasize the importance of an early implementation of these strategies in children and adolescents with obesity and, above all, to intensify the periodic controls in the boys due to its high resistance to treatment.

## Data Availability

The datasets generated during and/or analysed during the current study are available from the corresponding author on reasonable request.
